# Anthropomorphic tendencies in autism: A conceptual replication and extension of White and Remington (2019) and preliminary development of a novel anthropomorphism measure

**DOI:** 10.1177/13623613211039387

**Published:** 2021-09-18

**Authors:** Rachel A Clutterbuck, Punit Shah, Hok Sze Leung, Mitchell J Callan, Natalia Gjersoe, Lucy A Livingston

**Affiliations:** 1 University of Bath, UK; 2 Cardiff University, UK; 3 King’s College London, UK

**Keywords:** anthropomorphism, autism, personification, social cognition, theory of mind

## Abstract

White and Remington (2019) found that autistic people may have increased anthropomorphic tendencies to ascribe human-like attributes to non-human agents. However, it was unclear from their study whether this relationship holds after accounting for socio-demographic variables known to be associated with anthropomorphism. The psychometric properties of the anthropomorphism questionnaire they used has also not been investigated, raising concerns about whether it measures the same construct in people with differing levels of autistic traits. Addressing these issues, we re-examined the relationship between autism and anthropomorphism in a large sample of adults (*N* = 492). Conceptually replicating White and Remington, we found that autistic traits were significantly associated with greater anthropomorphic tendencies, even after accounting for age and sex (Study 1). Equally, psychometric concerns with the anthropomorphism questionnaire were revealed, leading us to refine this measure and re-analyse the data. A less clear-cut but significant association between autistic traits and anthropomorphism was found (Study 2). Our refined anthropomorphism measure also had improved psychometric properties, particularly showing that it is suitable for future autism research. Our findings are discussed in relation to individual differences in social-cognitive processing and we outline future directions for investigating mechanisms linking anthropomorphism and social cognition in autism.

**Lay abstract:**

Anthropomorphism is the tendency to attribute human-like qualities (e.g. thoughts and feelings) to non-human entities (e.g. objects and weather systems). Research by White and Remington (2019) suggested that anthropomorphism is more common in autistic compared to neurotypical adults, which is interesting given that autistic individuals sometimes misunderstand the thoughts and feelings of other people. In this article, we re-examined the link between autism and anthropomorphism in a large sample of adults with varying degrees of autistic traits, with several important methodological advances on previous research. Across two studies, we found that individuals with more autistic traits reported greater anthropomorphic tendencies. As part of these analyses, we had to develop a new, refined measure of anthropomorphism, which showed better reliability and validity than the original measure. This measure will be useful in future autism-related research. Overall, advancing White and Remington’s study, these findings help us to better understand individual differences in socially relevant processes, including those that may be enhanced in autism (e.g. anthropomorphism).

## Introduction

Anthropomorphism is the tendency to ascribe human-like attributes, such as mental states, to non-human agents. Researchers have theorised that anthropomorphism requires Theory of Mind (ToM), that is, the ability to represent the mental states of other people (e.g. Waytz et al., 2010). Interestingly, however, the notion that ToM is a prerequisite for anthropomorphism is not consistent with findings from autism research. Exploring the link between autism and anthropomorphism has potential to improve understanding of anthropomorphism, as well as socially relevant abilities of autistic and non-autistic people (see Atherton & Cross, 2018).

Autistic adults often perform atypically on ToM tasks requiring them to reason about the mental states of other humans or human-like agents (e.g. Livingston et al., 2019), which is linked to social difficulties in the real-world (Sasson et al., 2020). Yet, when reasoning about *non-human* agents, autistic people have often shown a similar or greater tendency to attribute mental states compared to non-autistic people. For example, Atherton and Cross (2019) compared participants’ performance on a classical Faux Pas ToM task (Stone et al., 1998) to an ‘anthropomorphised version’ of the task, whereby human characters and contexts were replaced with non-human equivalents. They found higher levels of autistic traits predicted poorer performance on the human Faux Pas task, as expected, but found no significant relationship between autistic traits and performance on the anthropomorphised version of the task. This indicated that, despite ToM atypicalities, autistic individuals may show a similar propensity to attribute mental states to non-human agents as non-autistic people. In contrast, other research has shown that autistic people have difficulty attributing human-like characteristics to animated geometric shapes (e.g. Klin, 2000), thus suggesting autistic people may have an atypically low tendency for anthropomorphic reasoning. However, most of these previous studies did not use tasks designed specifically to capture anthropomorphism, making it difficult to draw clear conclusions.

Encouragingly, recent studies have directly investigated autism-related anthropomorphic tendencies using validated measures. Tahiroglu and Taylor’s (2019) study, using self-report questionnaire measures, found a positive association between autistic traits and anthropomorphic tendencies. However, the low and limited range of anthropomorphism scores, as noted by the authors, raised concerns about the representativeness of the study’s student sample, making it difficult to judge the generalisability and overall robustness of these findings. In addition, the anthropomorphism measure used in this study only concerned current (adult) anthropomorphic beliefs, without consideration of potentially important childhood anthropomorphic tendencies (see Neave et al., 2015). Given that anthropomorphism is thought to be more common in childhood than adulthood (Epley et al., 2007), it is important to consider the differential links of childhood versus adult anthropomorphic tendencies with autism.

Addressing some of these issues, White and Remington (2019) compared autistic and non-autistic adults (*N* = 350) using the Anthropomorphism Questionnaire, which measures self-reported adult and childhood anthropomorphic tendencies (Neave et al., 2015). They found that autistic participants reported greater overall (*d* = 0.27) and adult anthropomorphism (*d* = 0.45) than non-autistic participants, but there was no significant group difference in the recollection of childhood anthropomorphism (*d* = 0.09). These findings indicated that anthropomorphic tendencies are potentially enhanced in autism, and the authors offered several intriguing explanations for this; for example, autistic people may anthropomorphise to manage loneliness and reduce uncertainty in their environment. Despite our enthusiasm for White and Remington’s important study, we note some outstanding questions arising from their research.

First, there is a general need for additional research on autism-related anthropomorphic tendencies using well-validated measures to corroborate previous findings. Apparent methodological strengths in White and Remington’s (2019) research provided an ideal starting point and the impetus to perform a *conceptual* replication of their work to test the robustness of the putative link between autism and anthropomorphism. Conceptual replications, which test the same fundamental hypothesis or idea as the original work, but with some methodological alterations, are arguably more informative than direct replications for testing the robustness of a phenomenon (Crandall & Sherman, 2016) and are increasingly found in autism research (e.g. Rodgers et al., 2018). Here – using the same questionnaire measures as White and Remington (2019) – instead of a case–control study, we measured autistic traits on a continuous scale in the general population, which is a widely used method to inform understanding of autism (see Happé & Frith, 2020). Furthermore, following growing awareness of the importance of replication efforts in (clinical) psychological science (see Tackett et al., 2017), we designed this study with replication in mind. Analyses were pre-registered and data are openly accessible, thereby improving upon the rigour of previous autism-related anthropomorphism research.

A second issue is that the proposed two-factor structure of the Anthropomorphism Questionnaire (i.e. Neave et al., 2015), used by White and Remington, has never been confirmed and therefore requires investigation. Furthermore, the Anthropomorphism Questionnaire has rarely been used in autism-related research and it is unknown if it measures the same construct (i.e. has measurement invariance) in people with differing levels of autistic traits. By examining the factor structure and measurement invariance of the Anthropomorphism Questionnaire, this study aimed to test the appropriateness of its use by White and Remington and in future autism-related research.

Third, sex differences in anthropomorphism are widely reported, with females showing greater anthropomorphic tendencies than males (e.g. Neave et al., 2015; Tahiroglu & Taylor, 2019; White & Remington, 2019). Age-related differences in anthropomorphism have also been reported in both child (Conrad et al., 2020) and adult (Neave et al., 2015) populations. Indeed, White and Remington reported a sex difference in anthropomorphism, but they did not control for this, or age, in statistical analyses when investigating the link between anthropomorphism and autism. This leaves open the possibility that observed relationships between autism and anthropomorphism may be confounded – and potentially underestimated – by age and/or sex-related differences in anthropomorphism. Moreover, given the uneven male-to-female sex ratio in autism (e.g. Lai & Szatmari, 2020), understanding the relative contributions of participant sex and autism to anthropomorphism merits particular investigation.

This study aimed to conceptually replicate White & Remington’s study while addressing the outstanding questions emerging from recent research. Specifically, we administered the same measures of autistic traits and anthropomorphism used by White and Remington and examined if the anthropomorphism questionnaire was invariant to high versus low levels of autistic traits. Furthermore, instead of sampling from a clinically diagnosed autism population, we aimed to test the unique association between autistic traits and anthropomorphism in a large sample from the general population, thereby enabling a well-powered investigation of this relationship, while giving us good statistical power to account for participant age and sex. Following White and Remington’s findings, we hypothesised that autistic traits would predict greater anthropomorphic tendencies.

## Methods

### Participants

Five-hundred adults from the UK general population were recruited online via *Prolific*. Eight participants were excluded for failing either one of two attention checks embedded into the study (e.g. ‘Please select “agree” to show you are reading this question’), whereby a failure to select the correct response was indicative that the participant may not be accurately reading the questions. One participant did not report their sex, so was excluded from any analyses involving this variable. This yielded a final sample of 492 adults aged 18–73 years (see Table 1 for descriptive statistics). This gave us at least 80% power to detect small associations in multivariate analysis (*f*^2^ = 0.02, *α* = 0.05). Participants gave informed consent and were debriefed following completion of the study. The study was approved by the local ethics committee.

**Table 1.** Participant characteristics and descriptive statistics – Studies 1 and 2.

	*n*	Age	Autistic traits	Anthropomorphism		
				Overall	Adult	Childhood
Female	369	32.31 (10.70)	3.11 (1.86)	37.14 (21.80)	14.91 (11.72)	22.22 (13.85)
Male	122	32.48 (12.13)	4.03 (2.06)	29.53 (23.43)	12.95 (12.09)	16.57 (14.11)
Low AQ-10	430	32.80 (11.17)	2.81 (1.40)	34.41 (21.92)	14.00 (11.52)	20.42 (13.93)
High AQ-10	62	29.13 (9.77)	7.00 (1.09)	40.71 (25.19)	17.36 (13.41)	23.36 (15.28)
Total	492	32.34 (11.06)	3.34 (1.95)	35.21 (22.43)	14.42 (11.82)	20.79 (14.12)

Table reports mean values with standard deviations in parentheses. Autistic traits were measured using the Autism-Spectrum Quotient-10 (AQ-10). Anthropomorphism was measured using the Anthropomorphism Questionnaire and scores were calculated as an overall score (range = 0–120) and for each subscale: adult and childhood (range = 0–60). Low AQ-10 (coded as 0; 331 females, 98 males and 1 missing sex datum) = AQ-10 scores < 6. High AQ-10 (coded as 1; 38 females and 24 males) = AQ-10 scores ⩾ 6. Participant sex was coded as males = 1 and females = 0.

### Materials and procedure

Participants completed questions about their age and sex followed by two questionnaires in a randomised order: The Autism-Spectrum Quotient-10 (AQ-10; Allison et al., 2012) and the Anthropomorphism Questionnaire (Neave et al., 2015). The AQ-10 measures autistic traits on a scale from 0 to 10. Participants respond to 10 questions (e.g. ‘I find it difficult to work out people’s intentions’) using a 4-point Likert-type scale from *Definitely Disagree* to *Definitely Agree*, with six reverse-worded items. Items are then scored in a binary format (0, 1). It is widely used in autism research (e.g. Livingston et al., 2020) and clinical practice (National Institute for Health and Care Excellence [NICE], 2012). The recommended AQ-10 cut-off for diagnostic referral (⩾ 6) has good sensitivity (0.88) and specificity (0.91) and the measure has previously been found to have good reliability (*α* > 0.85; Allison et al., 2012). Despite ongoing concerns regarding the psychometric properties of the AQ-10 (Taylor et al., 2020), the replication nature of our research and the good sensitivity and specificity of the AQ-10 cut-off – which remains valuable in classifying high/low autistic traits groups in general population samples – meant that the AQ-10 was the optimal measure of autistic traits in our research.

The 20-item Anthropomorphism Questionnaire measures individual differences in current (adult) and childhood anthropomorphic tendencies. Participants respond to questions like ‘On occasions I feel that my computer/printer is being deliberately awkward’ on a scale from 0 (*Not at All*) to 6 (*Very Much So*). Scores ranging from 0 to 60 are calculated for each of the two subscales of the measure, *childhood* and *adult* anthropomorphism, as well as an *overall* score ranging from 0 to 120. The subscales of the Anthropomorphism Questionnaire have previously been found to be correlated (*r* = 0.42) and the measure has good internal consistency (childhood subscale, *α* = 0.91; adult subscale, *α* = 0.86; Neave et al., 2015).

### Community involvement

This study was inspired by autistic students who highlighted White and Remington’s (2019) study as being of particular interest to them. They were overwhelmingly positive about the study as it resonated with their lived experiences. Equally, they highlighted some of the abovementioned limitations of previous research, including sex differences in anthropomorphism and the lack of replication of the findings. Together, this led to the co-development of this study, with a focus on accounting for participant sex in the analysis and replicating previous research.

## Study 1: replication of White and Remington (2019)

Analyses were pre-registered and data are openly accessible (see Supplemental Materials). Analyses were performed using R-3.5.3, JASP 0.12.2.0 and SPSS version 25. Detailed information about the statistical methods in the current studies is reported in the Supplemental Materials.

### Results

#### Reliability and factor analyses

In line with Sijtsma’s (2009) recommendations, multiple statistics are reported for internal consistency. Cronbach’s alpha (α) is the most widely used internal consistency statistic and is therefore reported in its standardised form to enable comparisons with existing research. McDonald’s omega (ω) statistic is reported because it is less biased than α (Trizano-Hermosilla & Alvarado, 2016). Additionally, the Greatest Lower Bound (glb) statistic is reported for its greater sensitivity to the number of scale items (Sijtsma, 2009) and robustness to violations of normality (Trizano-Hermosilla & Alvarado, 2016). α has typically been interpreted using guidelines offered by Nunnally (1978), which suggest that α values greater than 0.70 are acceptable. Although there are no conventional guidelines for ω and glb, they range from 0 to 1, with higher values generally indicating better reliability. These statistics are therefore interpreted in the same way as α in the present studies (Eldesouky & English, 2018; Rimkeviciene et al., 2016; Stinchfield et al., 2017). Internal consistency of all measures was in line with previous research: overall anthropomorphism (α_*standardised*_ = 0.91, *ω* = 0.91, glb = 0.96), adult anthropomorphism (α_*standardised*_ = 0.87, *ω* = 0.87, glb = 0.90), childhood anthropomorphism (α_*standardised*_ = 0.90, *ω* = 0.90, glb = 0.94) and AQ-10 (α_*standardised*_ = 0.57, *ω* = 0.60, glb = 0.73). Confirmatory Factor Analyses (CFAs) confirmed that a two-factor solution of the Anthropomorphism Questionnaire (*χ*^2^(169) = 1155.95, *p* < 0.001; comparative fit index (CFI) = 0.80; Tucker-Lewis index (TLI) = 0.77; root mean square error of approximation (RMSEA) = 0.11; standardised root mean square residual (SRMR) = 0.10) was a better fit than a one-factor solution (*χ*^2^(170) = 1960.68, *p* < 0.001; CFI = 0.63; TLI = 0.59; RMSEA = 0.15; SRMR = 0.12; Supplemental Table 1), supporting the proposed two-subscale structure of the measure. Notably, however, several model fit indices of both models were outside of the critical range (see Supplemental Materials – *Factor Analysis*).

Measurement invariance analysis (e.g. via Multi-Group CFAs) tests whether the same construct is being measured across different groups and is important for studying group differences on a measure (see Supplemental Materials – *Measurement Invariance*). To test measurement invariance of the Anthropomorphism Questionnaire, participants were split into groups of high (AQ-10 ⩾ 6, *n* = 62) and low (AQ-10 < 6, *n* = 430) autistic traits. Configural fit indices were not within the critical range, suggesting non-invariance of the Anthropomorphism Questionnaire to autistic traits (Supplemental Table 2), and therefore that the measure may not be measuring the same construct in people with high and low autistic traits. To guard against potential concerns with using the clinical AQ-10 cut-off, we also tested measurement invariance after grouping participants based on the median AQ-10 score, which revealed a similar pattern of results (Supplemental Table 2). Despite the poor model fit indices and non-invariance of the Anthropomorphism Questionnaire, we continued with our pre-registered correlational and multiple regression analyses to enable comparisons with White and Remington (2019). These issues are later addressed in Study 2.

#### Correlational and regression analyses

Replicating the pattern of results reported by White and Remington, autistic traits, as categorical and continuous variables, were positively correlated with greater overall and adult anthropomorphism, but not childhood anthropomorphism (Supplemental Table 3). Statistical comparisons of these effect sizes to White and Remington (2019) revealed no significant differences (Supplemental Table 4), indicating a replication of their main analyses. In addition, participant age was negatively correlated with childhood anthropomorphism and autistic traits, and sex was significantly associated with overall and childhood anthropomorphism, as well as autistic traits. Females reported greater levels of anthropomorphism and fewer autistic traits than males. These correlations confirmed the need to account for age and sex when examining the link between autistic traits and anthropomorphism in the multivariate analyses.

Multiple regression tested whether autistic traits, as categorical and continuous variables, predicted overall, adult and childhood anthropomorphism, while accounting for age and sex (Table 2). Following White and Remington, we found that autistic traits, operationalised as a categorical variable, uniquely predicted overall (*sr*^2^ = 0.01, *p* = 0.021) and adult anthropomorphism (*sr*^2^ = 0.01, *p* = 0.026), but not childhood anthropomorphism (*sr*^2^ = 0.01, *p* = 0.073), after controlling for age and sex. In addition, autistic traits, operationalised as a continuous variable, significantly predicted overall anthropomorphism (*sr*^2^ = 0.02, *p* = 0.003) and anthropomorphic tendencies in *both* adulthood (*sr*^2^ = 0.02, *p* = 0.003) and childhood (*sr*^2^ = 0.01, *p* = 0.020), after controlling for age and sex.

**Table 2.** Multiple regression analyses of the associations between autistic traits and overall, adult and childhood anthropomorphism – Study 1.

Main predictor	Model	*B*	SE *B*	*β*	*t*	*p*	*sr* ^2^	95% BCa CI	
								Lower	Upper
Autistic traits (categorical)	1. Overall anthropomorphism – *F*(3, 487) = 6.57, *p* < 0.001, *R*^2^ = 0.039								
	Age	−0.14	0.09	−0.07	−1.58	0.114	0.01	−0.32	0.06
	Sex	−8.25	2.32	−0.16	−3.55	<0.001	0.03	−12.57	−3.44
	**Autistic traits**	**7.03**	**3.04**	**0.10**	**2.31**	**0.021**	**0.01**	**0.78**	**13.68**
	2. Adult anthropomorphism – *F*(3, 487) = 2.74, *p* = 0.043, *R*^2^ = 0.017								
	Age	−0.03	0.05	−0.03	−0.57	0.572	0.00	−0.12	0.07
	Sex	−2.30	1.24	−0.08	−1.86	0.064	0.01	−4.53	0.16
	**Autistic traits**	**3.62**	**1.62**	**0.10**	**2.23**	**0.026**	**0.01**	**0.29**	**7.37**
	3. Childhood anthropomorphism – *F*(3, 487) = 7.88, *p* < 0.001, *R*^2^ = 0.046								
	Age	−0.12	0.06	−0.09	−2.04	0.042	0.01	−0.23	0.01
	Sex	−5.95	1.46	−0.18	−4.09	<0.001	0.03	−8.83	−2.75
	**Autistic traits**	**3.42**	**1.91**	**0.08**	**1.79**	**0.073**	**0.01**	**−0.58**	**7.42**
Autistic traits (continuous)	1. Overall anthropomorphism – *F*(3, 487) = 7.90, *p* < 0.001, *R*^2^ = 0.046								
	Age	−0.12	0.09	−0.06	−1.35	0.178	0.00	−0.31	0.07
	Sex	−9.05	2.35	−0.18	−3.86	<0.001	0.03	−13.68	−4.02
	**Autistic traits**	**1.60**	**0.53**	**0.14**	**3.04**	**0.003**	**0.02**	**0.52**	**2.62**
	2. Adult anthropomorphism – *F*(3, 487) = 3.96, *p* = 0.008, *R*^2^ = 0.024								
	Age	−0.02	0.05	−0.02	−0.34	0.734	0.00	−0.11	0.09
	Sex	−2.72	1.25	−0.10	−2.17	0.030	0.01	−4.96	−0.27
	**Autistic traits**	**0.83**	**0.28**	**0.14**	**2.94**	**0.003**	**0.02**	**0.27**	**1.42**
	3. Childhood anthropomorphism – *F*(3, 487) = 8.66, *p* < 0.001, *R*^2^ = 0.051								
	Age	−0.11	0.06	−0.08	−1.86	0.064	0.01	−0.23	0.02
	Sex	−6.34	1.47	−0.19	−4.30	<0.001	0.04	−9.09	−3.16
	**Autistic traits**	**0.78**	**0.33**	**0.11**	**2.34**	**0.020**	**0.01**	**0.09**	**1.44**

SE: standard error.

Sex was coded as males = 1 and females = 0. Autistic traits were measured using the Autism-Spectrum Quotient-10 (AQ-10). Anthropomorphism was measured using the Anthropomorphism Questionnaire with scores calculated as a total score (overall) and for each subscale (adult and childhood). For the autistic traits variable, participants were categorised into the high-AQ-10 = 1 or low-AQ-10 = 0 groups based on the AQ-10 cut-off. Autistic traits as a continuous measure were based on AQ-10 scores between 0 and 10. 95% bootstrapped bias-corrected and accelerated confidence intervals (95% BCa CI) with 2000 resamples are reported. The main predictor of each regression is highlighted in bold font.

It was also confirmed that the combined addition of participant age and sex improved model fit, over and above autistic traits, in predicting overall and childhood anthropomorphism.^1^ This was found when autistic traits were operationalised as categorical (overall anthropomorphism, ∆*R*^2^ = 0.030, ∆*F* = 7.68, *p* = 0.001; childhood anthropomorphism, ∆*R*^2^ = 0.042, ∆*F* = 10.62, *p* < 0.001) and continuous (overall anthropomorphism, ∆*R*^2^ = 0.034, ∆*F* = 8.58, *p* < 0.001; childhood anthropomorphism, ∆*R*^2^ = 0.044, ∆*F* = 11.33, *p* < 0.001) variables. Age and sex contributed less in the models predicting adult anthropomorphism (autistic traits [categorical], ∆*R*^2^ = 0.008, ∆*F* = 1.90, *p* = 0.150; autistic traits [continuous], ∆*R*^2^ = 0.010, ∆*F* = 2.45, *p* = 0.088; see Supplemental Table 5).

These analyses highlighted that sex was the strongest predictor of anthropomorphic tendencies across most of the regression models, even after accounting for autistic traits. There was also a sex difference in autistic traits. In view of research suggesting the possibility of a distinct female phenotype of autism (e.g. Lai & Szatmari, 2020), these results gave us reason to explore if sex moderated the relationship between autistic traits and anthropomorphism. However, repeating our regression analyses with the inclusion of the sex × autistic traits interaction revealed no such relationship (all *p*s > 0.05), while the original pattern of significance was found in relation to autistic traits and anthropomorphism (Supplemental Table 6).^1^

### Discussion

Correlational analyses conceptually replicated White and Remington’s results. We found the same pattern of statistical significance and effect sizes in the same direction as White and Remington; autistic traits were positively correlated with overall and adult anthropomorphism, but not childhood anthropomorphism. There were also no significant differences found between corresponding effect sizes in our analyses and White and Remington’s. In addition, following previous literature (e.g. Neave et al., 2015), females reported more anthropomorphic tendencies than males.

Multiple regression analyses, controlling for age and sex, revealed the same pattern of results as the correlations, with one notable difference. When autistic traits were operationalised as a continuous variable, they *did* significantly predict childhood anthropomorphism. This underscores the importance of controlling for age and sex, which was not done by White and Remington. The fact that the variance explained in most of the models was significantly increased when these socio-demographic variables were included as predictors also reaffirms this. Finally, although sex was the clearest predictor of anthropomorphism in most analyses, we showed that sex did not moderate the relationship between autistic traits and anthropomorphism, that is, the direction or strength of this relationship does not differ between males and females.

The main issue arising from Study 1 was the poor one- and two-factor model fit of the Anthropomorphism Questionnaire. This was the first CFA that has to our knowledge been performed on this measure, raising concerns about its factorial validity. Study 1 also revealed that the Anthropomorphism Questionnaire was not invariant to high/low levels of autistic traits, undermining our results and casting doubt on the robustness of White and Remington’s findings. Seemingly contradictory results between the poor factorial validity and the excellent internal consistency of the Anthropomorphism Questionnaire potentially indicate that the measure’s items form distinct but related factors (see Schmitt, 1996).

In Study 2, we performed Exploratory Factor Analysis (EFA) on the Anthropomorphism Questionnaire, with the aim of refining the measure and improving its factorial validity to conduct a more robust analysis of our data. Furthermore, it has been proposed that configural fit indices are influenced by the model fit of the measure (e.g. Jorgensen, 2017). Therefore, by improving the factor structure of the questionnaire, it was possible that measurement invariance to autistic traits may be achieved. Overall, in Study 2, we aimed to refine the Anthropomorphism Questionnaire, and subsequently re-test the link between autistic traits and anthropomorphism.

## Study 2: anthropomorphism questionnaire refinement and re-analysis

### Anthropomorphism questionnaire refinement: factor analyses and internal consistency

To improve the Anthropomorphism Questionnaire, the data set from Study 1 (*N* = 492) was halved, with 246 participants in each set. The first set of data was used to perform EFA on the original 20-item Anthropomorphism Questionnaire. This revealed two correlated factors (*r* = 0.47) with eigenvalues greater than 1 (Supplemental Table 7). Based on these results, a stepwise process of removing items with the lowest factor loadings was used to improve model fit, as is often done to refine measures (e.g. Matsunaga, 2010). The lowest loading items were removed one-by-one until the fit indices were within the critical range. This enabled us to retain the maximum number of items while moving towards a better factor structure of the refined measure.

Accordingly, CFA of the second set of data showed that nine items of the Anthropomorphism Questionnaire (four on the *adult* subscale and five on the *childhood* subscale) formed a two-factor structure with fit indices within or close to the acceptable range (*χ*^2^(26) = 74.02, *p* < 0.001; CFI = 0.95; TLI = 0.93; RMSEA = 0.09; SRMR = 0.04, Table 3). Further item removal worsened the fit indices of the two-factor model, so this nine-item version was the optimal solution for the refined measure. CFA of a one-factor solution of the nine items revealed fit indices outside the critical range (*χ*^2^(27) = 312.78, *p* < 0.001; CFI = 0.71; TLI = 0.61; RMSEA = 0.21; SRMR = 0.16, Table 3). Finally, re-analysis of the entire data set (*N* = 492), drawing on participants’ responses to just these nine items, revealed two correlated factors (*r* = 0.30) with eigenvalues greater than 1 (Supplemental Table 8). CFA revealed an acceptable two-factor model fit (*χ*^2^(26) = 136.26, *p* < 0.001; CFI = 0.95; TLI = 0.93; RMSEA = 0.09; SRMR = 0.04) with moderately correlated factors (*r* = 0.27), but a poor one-factor model fit (*χ*^2^(27) = 693.23, *p* < 0.001; CFI = 0.69; TLI = 0.58; RMSEA = 0.22; SRMR = 0.17).

**Table 3.** Factor loadings for the nine-item Anthropomorphism Questionnaire – Study 2.

Item	CFA (two-factor)		CFA (one-factor)
	Factor 1	Factor 2	
I sometimes wonder if my computer deliberately runs more slowly after I have shouted at it (1)	0.68	–	0.17
On occasions I feel that my computer/printer is being deliberately awkward (5)	0.81	–	0.21
On occasion I feel that the weather conditions are being deliberately bad in order to ruin a social event (7)	0.58	–	0.10
I sometimes think that if my computer/printer is made to feel happy and/or wanted, then they will be less likely to malfunction (14)	0.65	–	0.16
When I was a child I always made sure my favourite toy was comfortable (e.g. sitting up or tucked into bed) when I left the room (2)	–	0.82	0.81
As a child I sometimes said ‘hello’ and ‘good night’ to some of my favourite toys (3)	–	0.82	0.82
As a child, when I put away my toys I made sure that any odd ones lying around were placed with the others so that they would not feel lonely (8)	–	0.87	0.87
If I threw out a toy when I was a child I worried that it might think I had rejected it (10)	–	0.67	0.68
When I was a child, I made sure that when I put my toys away the ones who were friends were placed side by side (20)	–	0.77	0.77

CFA: Confirmatory Factor Analysis; Factor 1: adult anthropomorphism subscale; Factor 2: childhood anthropomorphism subscale.

*N* = 246. Anthropomorphism Questionnaire item numbers are in parentheses.

CFA (two-factor) model revealed a moderate correlation between factors (*r* = 0.21).

In contrast to Study 1, multi-group CFAs showed that the nine-item Anthropomorphism Questionnaire was invariant to level of autistic traits when participants were grouped using the AQ-10 clinical cut-off (⩾ 6) and the AQ-10 median (> 3). Most of the configural fit indices were within or very close to the critical range and ΔCFI was < 0.01 at the metric, scalar and strict levels of invariance (Supplemental Table 9). The 7-point Likert-type scale, measuring participants’ level of agreement to the nine statements, from 0 – *(Not at all)* to 6 – *(Very much so)*, was unchanged from the original Anthropomorphism Questionnaire (Neave et al., 2015). The overall scale and subscales of the newly refined nine-item measure had good internal consistency (overall anthropomorphism, α_*standardised*_ = 0.82, *ω* = 0.83, glb = 0.91; adult anthropomorphism, α_*standardised*_ = 0.80, *ω* = 0.81, glb = 0.83; childhood anthropomorphism, α_*standardised*_ = 0.90, *ω* = 0.90, glb = 0.92). Supplemental Table 10 presents descriptive statistics.

### Regression analyses

Using the psychometrically improved nine-item anthropomorphism measure, we re-examined the association between autistic traits and anthropomorphism using regression analysis with predictors that had been significantly linked to anthropomorphism in Study 1 (see Table 4). Autistic traits, when operationalised as a categorical variable, did not significantly predict overall (*sr*^2^ = 0.01, *p* = 0.088), adult (*sr*^2^ = 0.01, *p* = 0.101) or childhood anthropomorphism (*sr*^2^ = 0.00, *p* = 0.236), after controlling for age and sex. In contrast, when autistic traits were operationalised as a continuous variable, the results were in line with Study 1 and replicated White and Remington. Autistic traits significantly predicted overall (*sr*^2^ = 0.01, *p* = 0.017), adult (*sr*^2^ = 0.01, *p* = 0.024), but not childhood (*sr*^2^ = 0.01, *p* = 0.091) anthropomorphism, after controlling for age and sex.

**Table 4.** Multiple regression analyses of the associations between autistic traits and overall, adult and childhood anthropomorphism, using the nine-item Anthropomorphism Questionnaire – Study 2.

Main predictor	Model	*B*	SE *B*	*β*	*t*	*p*	*sr* ^2^	95% BCa CI	
								Lower	Upper
Autistic traits (categorical)	1. Overall anthropomorphism – *F*(3, 487) = 9.40, *p* < 0.001, *R*^2^ = 0.055								
	Age	−0.12	0.05	−0.12	−2.62	0.009	0.01	−0.22	−0.03
	Sex	−5.03	1.17	−0.19	−4.31	<0.001	0.04	−7.18	−2.61
	**Autistic traits**	**2.61**	**1.53**	**0.08**	**1.71**	**0.088**	**0.01**	**−0.34**	**5.70**
	2. Adult anthropomorphism – *F*(3, 487) = 2.49, *p* = 0.060, *R*^2^ = 0.015								
	Age	−0.03	0.02	−0.06	−1.25	0.211	0.00	−0.07	0.02
	Sex	−1.00	0.55	−0.08	−1.82	0.070	0.01	−1.99	0.05
	**Autistic traits**	**1.18**	**0.72**	**0.08**	**1.64**	**0.101**	**0.01**	**−0.25**	**2.76**
	3. Childhood anthropomorphism – *F*(3, 487) = 9.06, *p* < 0.001, *R*^2^ = 0.053								
	Age	−0.09	0.04	−0.11	−2.57	0.010	0.01	−0.17	−0.02
	Sex	−4.04	0.92	−0.20	−4.38	<0.001	0.04	−5.72	−2.26
	**Autistic traits**	**1.43**	**1.21**	**0.05**	**1.19**	**0.236**	**0.00**	**−1.02**	**3.79**
Autistic traits (continuous)	1. Overall anthropomorphism – *F*(3, 487) = 10.40, *p* < 0.001, *R*^2^ = 0.060								
	Age	−0.11	0.05	−0.11	−2.42	0.016	0.01	−0.20	−0.02
	Sex	−5.37	1.18	−0.20	−4.55	<0.001	0.04	−7.60	−3.12
	**Autistic traits**	**0.64**	**0.27**	**0.11**	**2.40**	**0.017**	**0.01**	**0.11**	**1.13**
	2. Adult anthropomorphism – *F*(3, 487) = 3.31, *p* = 0.020, *R*^2^ = 0.020								
	Age	−0.02	0.02	−0.05	−1.07	0.286	0.00	−0.06	0.02
	Sex	−1.14	0.55	−0.10	−2.06	0.040	0.01	−2.13	−0.11
	**Autistic traits**	**0.28**	**0.13**	**0.11**	**2.26**	**0.024**	**0.01**	**0.06**	**0.55**
	3. Childhood anthropomorphism – *F*(3, 487) = 9.57, *p* < 0.001, *R*^2^ = 0.056								
	Age	−0.09	0.04	−0.11	−2.42	0.016	0.01	−0.16	−0.01
	Sex	−4.23	0.93	−0.20	−4.53	<0.001	0.04	−5.91	−2.42
	Autistic traits	**0.36**	**0.21**	**0.08**	**1.69**	**0.091**	**0.01**	**−0.08**	**0.82**

SE: standard error.

Sex was coded as males = 1 and females = 0. Autistic traits were measured using the Autism-Spectrum Quotient-10 (AQ-10). Anthropomorphism was measured using the nine-item Anthropomorphism Questionnaire with scores calculated as a total score (overall) and for each subscale (adult and child). For the autistic traits variable, participants were categorised into the high-AQ-10 = 1 or low-AQ-10 = 0 groups based on the AQ-10 cut-off. Autistic traits as a continuous trait measure were based on AQ-10 scores between 0 and 10. 95% bootstrapped bias-corrected and accelerated confidence intervals (95% BCa CI) with 2000 resamples are reported. The main predictor of each regression is highlighted in bold font.

### Discussion

A two-factor structure of the Anthropomorphism Ques-tionnaire was confirmed in Study 2, mitigating the most serious concerns about an altogether different factorial structure. Nonetheless, given the poor one- and two-factor model fit indices of the measure reported in Study 1, Study 2 introduced a psychometrically improved nine-item version of this measure. The nine-item Anthropomorphism Questionnaire had a relatively good two-factor structure, which was invariant to autistic traits. This suggests it measures the same construct in people with high and low levels of autistic traits and, when compared to the original measure, is more appropriate for use in future research on autism-related anthropomorphism. With that said, the CFA fit indices of the one-factor model for the nine-item Anthropomorphism Questionnaire were poor, so analyses using the overall anthropomorphism score should be interpreted cautiously. In this study, we reported overall anthropomorphism scores to make comparisons with Study 1 and White and Remington (2019), but future studies should consider using the individual subscales only.

The internal consistency of the nine-item Anthropo-morphism Questionnaire was excellent. While the internal consistency statistics were slightly lower for the nine-item Anthropomorphism Questionnaire than the original measure, this would be expected for a measure with fewer items. Given that the original measure had very high internal consistency statistics (e.g., glb = 0.90 – 0.94), which potentially indicated redundant items within the measure (e.g., Streiner, 2003), the slightly lower internal consistency statistics of the nine-item Anthropomorphism Questionnaire (e.g., glb = 0.83 – 0.92) may suggest that this refined version has greater independence between items.

Using this refined measure, a re-examination of our data showed that, when socio-demographic variables were controlled for, and when autistic traits were operationalised as a continuous variable, there was a significant relationship between autistic traits and overall and adult anthropomorphism, but not childhood anthropomorphism. Again, this conceptually replicated White and Remington’s findings. However, when autistic traits were operationalised as a categorical variable, there were no significant associations with anthropomorphism. This may be due to the loss of information from categorical versus continuous data and the consequent reduction in statistical power (cf. Altman & Royston, 2006).

## General discussion

This study examined the link between autistic traits and anthropomorphism, drawing on a large sample from the general population to enable well-powered statistical analyses. We initially conceptually replicated White and Remington’s (2019) finding that autistic traits positively predict overall anthropomorphism and adult anthropomorphic tendencies. The findings are also consistent with Tahiroglu and Taylor’s (2019) research, which found an association between autistic traits and anthropomorphism using alternative measures of these constructs. In addition, by conducting multivariate analyses not performed in previous research, we showed that the relationship between autistic traits and anthropomorphism holds after accounting for participant age and sex (Study 1). Yet, after developing and using an improved version of the Anthropomorphism Questionnaire in Study 2, there was a less clear-cut pattern of results compared to Study 1 and White and Remington (2019). Overall, the findings of the current research provide moderate support for a relationship between autistic traits and anthropomorphism. Moving forward, further conceptual and empirical research is warranted to ascertain why the positive link between autistic traits and anthropomorphism is not robust across different methods of measurement and statistical analysis, as shown in the current research. A re-analysis of White and Remington’s data using our refined nine-item measure may be a useful starting point for such research.

White and Remington (2019) proposed that the positive relationship between autistic traits and anthropomorphism speaks against ToM difficulties in autism, given the attribution of mental states to non-human agents (e.g. Atherton & Cross, 2018). However, a critical distinction between anthropomorphism and ToM is that anthropomorphism cannot be classed in terms of accuracy, whereas ToM can be. For example, as pens cannot have emotional states, it is impossible to measure how accurately an individual has inferred a pen’s emotions, whereas it *is* possible to measure how accurately an individual has inferred the emotions of another human. Corresponding with recent theory (see Sagiv et al., 2017), we propose that autistic people may have strong *tendencies* to attribute mental states as often, or even more often, than non-autistic people, which leads to indiscriminate attribution of mental states to people and objects (i.e. anthropomorphism) alike. Enhanced anthropomorphic tendencies may not necessarily transfer to accuracy in identifying people’s mental states (i.e. ToM). Another interpretation of these findings could be consideration of this phenomenon as an adaptive compensatory strategy (see Livingston et al., 2021). Engagement in pseudo-social interactions with non-human agents may help people with autistic traits to improve social interactions, despite ongoing social-cognitive difficulties (Livingston et al., 2020). Testing the inter-relationships between autism, anthropomorphism, social cognition and compensation in future research could shed light on these ideas.

Consistent with previous research (e.g. Neave et al., 2015), sex remained a uniquely strong predictor of overall and childhood anthropomorphism even after accounting for autistic traits in all multiple regression analyses, but sex did not moderate the relationship between autistic traits and anthropomorphism. Age was also a unique predictor of childhood and/or overall anthropomorphism across most regression analyses. The importance of accounting for these socio-demographic variables is emphasised by the increased amount of variance explained following their inclusion in most of the regression models. We therefore propose that including age and sex in analyses moving forward is essential to accurately quantify the unique contribution of autistic traits to anthropomorphic tendencies and social-cognitive abilities in autism more generally.

Recruiting a general population sample has enabled us to collect a uniquely large data set to perform well-powered statistical analyses (e.g. measurement invariance testing and multiple regression analyses), extending our understanding of the relationship between autism and anthropomorphism in a way that would not necessarily be feasible if using smaller samples from a clinically diagnosed autistic population. Sampling from the general population also allows access to individuals who have high autistic traits but may not have a formal diagnosis of autism. This approach is more inclusive of autistic compensators who, despite reporting high autistic traits, may be less likely to have a clinical diagnosis due to superior compensatory abilities (Livingston & Happé, 2017). Nevertheless, convenience sampling can limit the representativeness of research findings, and ultimately, our methods and analytic approaches would be further strengthened in a future study including diagnosed autistic people and dimensional measures of autistic behaviour (e.g. Lord et al., 2000).

There are some methodological limitations in the research and future directions worth noting. Our results cannot speak to why the relationships between autistic traits and anthropomorphism were different when autistic traits were operationalised as categorical versus continuous variables. This may be, for example, due to the unequal sample sizes between high/low levels of autistic traits as a consequence of our convenience sampling method. Future investigations would benefit from better matching the numbers of participants in high/low autistic traits groups. Similarly, future case–control studies should match socio-demographic factors such as age and sex, as well as other cognitive abilities, such as intelligence, which are known to be associated with social cognition (e.g. Morrison et al., 2019) and may therefore be important when investigating socially relevant processes like anthropomorphism.

We used the AQ-10 to measure autistic traits in accordance with White and Remington’s methods. The AQ-10 was also useful in creating groups to conduct measurement invariance testing, given the well-defined clinical cut-off. However, this measure has relatively low internal consistency, which is in line with recent evidence that the AQ-10 has a multi-dimensional factor structure and psychometric issues (Taylor et al., 2020). Clinical cut-offs for the AQ-10 have also been poorly applied in research and clinical practice (Waldren et al., 2021). Potential concerns with the use of the AQ-10 in this study are mitigated by the fact that we partly replicated Tahiroglu and Taylor’s (2019) study, which used a longer, arguably more appropriate measure of autistic traits. Nonetheless, measuring autistic traits with better psychometric properties to obviate emerging concerns with the AQ-10 is critical. More broadly, given that we found more obvious links with anthropomorphism when autistic traits were operationalised as a continuous variable, more sensitive measures of autistic traits and behaviours will likely be important in future autism-related anthropomorphism research.

Our use of the Anthropomorphism Questionnaire (Neave et al., 2015) instead of the Individual Differences in Anthropomorphism Questionnaire (IDAQ; Waytz et al., 2010) also warrants critical discussion. We propose that the Anthropomorphism Questionnaire may be a more conceptually appropriate measure of anthropomorphism, particularly in autistic populations, given its focus on concrete behaviours and beliefs, in contrast to the IDAQ, which requires a deeper understanding of abstract concepts. Nonetheless, our research has revealed that the original Anthropomorphism Questionnaire has a poor one- and two-factor structure, and the original measure is not invariant to level of autistic traits. This unfortunately poses a challenge to the validity of White and Remington’s findings as well as our results replicating their findings in Study 1.

To help overcome this issue, the current studies – given the large data set – presented a timely opportunity to advance the methods for measuring anthropomorphism. The nine-item Anthropomorphism Questionnaire developed in Study 2 was found to be a psychometrically superior measure of anthropomorphism to the original version. Importantly, it was invariant to differing levels of autistic traits, which means that we can be more confident of our results in Study 2. Our research was, however, not designed to develop a novel anthropomorphism measure. After being administered as a standalone instrument, it will require further psychometric development, which is often lacking in autism and social cognition research (see Clutterbuck et al., 2021). To test its construct validity, for example, future research could test its association with other measures of anthropomorphism, such as the IDAQ.

Following testing in large samples of autistic and non-autistic people, this nine-item measure has potential to address outstanding questions on the nature of the link between autism and anthropomorphism. Adapting this measure for use in children may also be possible. The current findings failed to show a clear-cut link between autistic traits and childhood anthropomorphism (as per White & Remington, 2019), when using both the 9- and 20-item measures. This may be due to a lack of statistical power, a genuine lack of a relationship, or perhaps the childhood subscale is limited as a retrospective measure. Future research investigating anthropomorphic tendencies in autistic children using an adapted version of our nine-item anthropomorphism questionnaire should inform much needed understanding of the development of any relationship between autistic traits and childhood anthropomorphism.

To summarise, this study broadly indicates that autistic traits are associated with greater overall anthropomorphic tendencies, in line with White and Remington (2019). Building on White and Remington’s study, this effect appears to be present after controlling for participant age and sex, and when using a psychometrically improved version of the anthropomorphism measure. Overall, this study mostly supports previous findings on a positive link between autistic traits and anthropomorphism, but also leaves some unanswered questions. Moving forward, it is hoped that our research will help to advance the measurement of anthropomorphism, in both autism and general psychological research, particularly in tackling outstanding questions we have noted on mechanisms linking autism, anthropomorphism and social cognition.

## Supplementary Material

Supplementary material

Supplementary material

## Notes

1. This was not pre-registered and we thank a reviewer for suggesting this analysis.
