# Supplementary material for: Anthropomorphic tendencies in autism: A conceptual replication and extension of White and Remington (2019) and preliminary development of a novel anthropomorphism measure
Source: Autism. 2021 Sep 18;26(4):940–50. doi: 10.1177/13623613211039387 (PMC9014771; doi:10.1177/13623613211039387)
Supplement: Supplementary material [file sj-docx-1-aut-10.1177_13623613211039387.docx]

**Supplemental Materials**

Clutterbuck, R. A., Shah, P., Leung, H. S., Callan, M. J., Gjersoe, N., & Livingston, L. A. (2021). Anthropomorphic tendencies in autism: A conceptual replication and extension of White and Remington (2019) and preliminary development of a novel anthropomorphism measure. *Autism*.

***Factor Analysis***

Exploratory Factor Analyses use principal axis factoring and Oblimin rotation. All Confirmatory Factor Analyses use Maximum Likelihood estimation. To establish model-fit of the factor analyses, we report multiple fit indices to reduce the risk of Type I and II errors (Hu & Bentler, 1999): CFI, TLI, SRMR, and RMSEA are reported as these are commonly reported SEM fit indices (e.g., Taasoobshirazi & Wang, 2016). Chi-squared statistics (χ^2^) are also reported, as a matter of good-practice, however given their sensitivity to large sample sizes, their significance level should be ignored (e.g., Barrett, 2007).

To examine model fit, we followed the widely used guidance recommended by Hu and Bentler (1999), which are considered to reduce Type II error rates, with acceptable levels of Type I error rates: A value close to or greater than .95 for TLI and CFI, a value close to or below .08 for SRMR and a value close to or below .06 for RMSEA.

***Measurement Invariance***

We used Multi-Group Factor Analyses (MCFAs) to establish measurement invariance of the Anthropomorphism Questionnaire in people with high and low levels of autistic traits based on a 2-factor model fit of the measure. MCFA is a stepwise factor analytic process which compares nested models of invariance with increasing levels of equality constraint across groups. A maximum of four levels of invariance are tested (configural, metric, scalar, strict) in line with common practice (Putnick & Borstein, 2016). Invariance of the configural model is established if the fit indices are within the critical range. Invariance of the remaining models – metric, scalar, and strict – is tested by comparing the change between nested models. Change in CFI is used to assess measurement invariance between nested models, which is the most widely reported method (Putnick & Borstein, 2016). Following the guidelines by Cheung and Rensvold (2002), change in CFI ≤ .01 indicates invariance between the models.

To perform MCFAs, participants were grouped based on the AQ-10 cut off (= 6). To mitigate any concerns about the AQ-10 cut-off, which may undermine the measurement invariance results, analyses were performed by grouping participants using the AQ-10 median in the sample (= 3). There are inherent limitations of using a cut-off value which is sample-specific, but this supplementary approach is designed to provide converging evidence and thereby allay potential concerns that certain readers may have with the established clinical AQ-10 cut-off (see also, Waldren et al., 2021).

***Multiple Regression Analyses***

VIF values across all multiple regression analyses suggested that multicollinearity was not a concern (all < 10). Durbin-Watson statistics indicated that residuals were uncorrelated (~ 2) and independent. Inspection of the Q-Q plots revealed a potential violation of normality of the residuals, hence we report 95% bias-corrected and accelerated bootstrapped confidence intervals throughout the current studies (BCa CIs; with 2000 resamples).

**Study 1**

**Supplemental Table 1**

*Factor Loadings from CFA of the Anthropomorphism Questionnaire – Study 1.*

|  | CFA (two-factor) | | CFA  (one-factor) |
| --- | --- | --- | --- |
| Item | Factor 1 | Factor 2 |  |
| I sometimes wonder if my computer deliberately runs more slowly after I have shouted at it (1) | .62 | -- | .38 |
| On occasions I feel that my computer/printer is being deliberately awkward (5) | .68 | -- | .45 |
| I sometimes wonder if my personal possessions appreciate it when I have given them a good clean (6) | .68 | -- | .58 |
| On occasion I feel that the weather conditions are being deliberately bad in order to ruin a social event (7) | .56 | -- | .34 |
| I do think that certain cars have a specific personality (9) | .62 | -- | .41 |
| If I accidentally break one of my favourite possessions I make sure that I apologise to it for my clumsiness (11) | .60 | -- | .63 |
| I think that some trees are friendly while others have an air of menace (13) | .61 | -- | .49 |
| I sometimes think that if my computer/printer is made to feel happy and/or wanted, then they will be less likely to malfunction (14) | .73 | -- | .49 |
| I sometimes feel that the sea can be angry (17) | .57 | -- | .47 |
| Part of the reason why I picked a new car/electrical item was because when I first saw it I felt that it had a friendly personality (19) | .59 | -- | .49 |
| When I was a child I always made sure my favourite toy was comfortable (e.g. sitting up or tucked into bed) when I left the room (2) | -- | .75 | .67 |
| As a child I sometimes said “hello” and “good night” to some of my favourite toys (3) | -- | .75 | .66 |
| When I was a child I held birthday parties for my favourite toys (4) | -- | .56 | .51 |
| As a child, when I put away my toys I made sure that any odd ones lying around were placed with the others so that they wouldn’t feel lonely (8) | -- | .84 | .75 |
| If I threw out a toy when I was a child I worried that it might think I had rejected it (10) | -- | .75 | .70 |
| As a child, I felt that some of my toys had become ill (12) | -- | .54 | .60 |
| As a child I felt at times that some of my toys were in a bad mood (15) | -- | .56 | .62 |
| As a child, the thought of how my favourite toys would cope without me if I died was something that I worried about (16) | -- | .56 | .61 |
| I sometimes wonder that if toys are stored out of sight in a dark attic or room, they might feel lonely or unloved (18) | -- | .74 | .76 |
| When I was a child, I made sure that when I put my toys away the ones who were friends were placed side by side (20) | -- | .82 | .76 |

*Note. N* = 492. Anthropomorphism Questionnaire item numbers are in parentheses. CFA = Confirmatory Factor Analysis. Factor 1 = adult subscale, Factor 2 = childhood subscale. CFA (two-factor) model showed a moderate correlation between factors (*r* = .52).

**Supplemental Table 2**

*Measurement Invariance of the Anthropomorphism Questionnaire to Level of Autistic Traits using the AQ-10 Cut-off and AQ-10 Median Split, with Multi-Group Confirmatory Factor Analysis – Study 1.*

| Grouping |  | χ² | df | CFI | TLI | RMSEA | SRMR | Decision |
| --- | --- | --- | --- | --- | --- | --- | --- | --- |
| AQ-10  Cut-off | Configural | 1373.92 | 338 | .787 | .760 | .112 | .093 | Reject |
| AQ-10 Median Split | Configural | 1396.86 | 338 | .783 | .756 | .113 | .096 | Reject |

*Note:* χ²: chi-square goodness of fit test; df: degrees of freedom; CFI: Comparative Fit Index; TLI: Tucker-Lewis Index; RMSEA: Root Mean Squared Error of Approximation; SRMR: Standardised Root Mean Squared Residual. AQ-10 = Autism-Spectrum Quotient-10. Analyses were performed using maximum likelihood estimation. Participants were categorised into the High AQ-10 = 1 or Low AQ-10 = 0 groups based on the AQ-10 cut-off of 6. For the Median Split MCFA analysis, participants were split into two groups based on the AQ-10 sample median of 3: > Median = 1 (*n* = 209), or ≤ Median = 0 (*n* = 283). Invariance at the configural level is determined if fit indices are within the critical range.

**Supplemental Table 3**

*Descriptive Statistics and Correlations – Study 1.*

| Measure | *M*(*SD*) | 1 | 2 | 3 | 4 | 5 | 6 | 7 |
| --- | --- | --- | --- | --- | --- | --- | --- | --- |
| 1. Age | 32.34(11.06) | – |  |  |  |  |  |  |
| 2. Sex | – | .01  [-.08, .10] | – |  |  |  |  |  |
| 3. Overall Anthropomorphism | 35.21(22.43) | -.08  [-.17, .01] | -.15*  [-.23, -.06] | – |  |  |  |  |
| 4. Adult Anthropomorphism | 14.42(11.82) | -.04  [-.12, .05] | -.07  [-.16, .02] | .84**  [.81, .86] | – |  |  |  |
| 5. Childhood Anthropomorphism | 20.79(14.12) | -.10*  [-.20, -.01] | -.17**  [-.26, -.09] | .89**  [.87, .91] | .49**  [.42, .56] | – |  |  |
| 6. Autistic Traits (Categorical) | – | -.11*  [-.18, -.03] | .12*  [.02, .22] | .09*  [-.00, .19] | .09*  [-.00, .19] | .07  [-.03, .16] | – |  |
| 7. Autistic Traits (Continuous) | 3.34(1.95) | -.16*  [-.24, -.07] | .20**  [.11, .29] | .11*  [.03, .20] | .12*  [.03, .21] | .08  [-.01, .17] | .72**  [.66, .76] | – |
| *Note.* Pearson’s *r* correlations are reported, with point-biserial correlations for binary variables. **p* < .05, ***p* < .001. 95% bootstrapped bias-corrected and accelerated confidence intervals (95% BCa CI) with 2000 resamples are in square brackets. Participant Sex was coded as males = 1, females = 0. Autistic traits were measured using the Autism-Spectrum Quotient-10 (AQ-10) and anthropomorphism was measured using the Anthropomorphism Questionnaire, with scores calculated as a total score (overall) and for each subscale (adult & child). For the categorical Autistic Traits variable, participants were categorised into the High AQ-10 = 1 or Low AQ-10 = 0 groups based on the AQ-10 cut-off (≥ 6). Autistic Traits as a continuous variable was based on AQ-10 scores between 0-10. | | | | | | | | |

**Supplemental Table 4**

*Statistical Comparison with White and Remington’s (2019) Effect Sizes – Study 1.*

| Independent Variable | Dependent Variable  (Anthropomorphism) | Effect Size (*r*) | | | NHST | |
| --- | --- | --- | --- | --- | --- | --- |
|  |  | Current | W&R | *z* | | *p* |
| **Autistic Traits (Categorical)** | Overall | .09 | .13 | -0.58 | | .564 |
|  | Adult | .09 | .22 | -1.90 | | .057 |
|  | Childhood | .07 | .05 | 0.29 | | .775 |
| **Autistic Traits (Continuous)** | Overall | .11 | .13 | -0.29 | | .773 |
|  | Adult | .12 | .22 | -1.47 | | .142 |
|  | Childhood | .08 | .05 | 0.43 | | .668 |

*Note. r* = Pearson’s *r* statistic. NHST = Null Hypothesis Significance Test. W&R = White and Remington (2019). Cohens *d* was converted to Pearson’s *r* using the ‘effectsize’ R package (Ben-Shachar et al., 2020) and NHST of the difference between the current and W&R’s effect sizes (*r*) were performed using the ‘cocor’ R package (Diedenhofen & Musch, 2015).

**Supplemental Table 5**

*Hierarchical Regression Analyses of the Associations between Autistic Traits and Overall, Adult, and Childhood Anthropomorphism – Study 1*.

| Main Predictor | Model | *B* | *SE B* | *β* | *t* | *p* | *sr^2^* | 95% BCa CI | |  |
| --- | --- | --- | --- | --- | --- | --- | --- | --- | --- | --- |
|  |  |  |  |  |  |  |  | Lower | Upper |  |
| **Autistic Traits**  (Categorical) | **Overall Anthropomorphism** | | | | | | | | |  |
|  | Step 1 — *F* (1, 489) = 4.24, *p* = .04, *R^2^* = .009 | | | | | | | | |  |
|  | Autistic Traits | 6.26 | 3.04 | .09 | 2.06 | .040 | .01 | 0.08 | 12.91 |  |
|  | Step 2 — *F* (3, 487) = 6.57, *p* < .001, *R^2^* = .039 | | | | | | | | |  |
|  | Age | -0.14 | 0.09 | -.07 | -1.58 | .114 | .01 | -0.33 | 0.04 |  |
|  | Sex | -8.25 | 2.32 | -.16 | -3.55 | <.001 | .03 | -12.70 | -3.09 |  |
|  | Autistic Traits | 7.03 | 3.04 | .10 | 2.31 | .021 | .01 | 0.85 | 13.83 |  |
|  | **Adult Anthropomorphism** | | | | | | | | |  |
|  | Step 1 — *F* (1, 489) = 4.38, *p* = .037, *R^2^* = .009 | | | | | | | | |  |
|  | Autistic Traits | 3.35 | 1.60 | .09 | 2.09 | .037 | .01 | -0.06 | 7.36 |  |
|  | Step 2 — *F* (3, 487) = 2.74, *p* = .043, *R^2^* = .017 | | | | | | | | |  |
|  | Age | -0.03 | 0.05 | -.03 | -0.57 | .572 | .00 | -0.12 | 0.08 |  |
|  | Sex | -2.30 | 1.24 | -.08 | -1.86 | .064 | .01 | -4.74 | 0.15 |  |
|  | Autistic Traits | 3.62 | 1.62 | .10 | 2.23 | .026 | .01 | 0.50 | 7.20 |  |
|  | **Childhood Anthropomorphism** | | | | | | | | |  |
|  | Step 1 — *F* (1, 489) = 2.30, *p* = .130, *R^2^* = .005 | | | | | | | | |  |
|  | Autistic Traits | 2.90 | 1.92 | .07 | 1.52 | .130 | .01 | -0.99 | 6.72 |  |
|  | Step 2 — *F* (3, 487) = 7.88, *p* < .001, *R^2^* = .046 | | | | | | | | |  |
|  | Age | -0.12 | 0.06 | -.09 | -2.04 | .042 | .01 | -0.24 | -0.00 |  |
|  | Sex | -5.95 | 1.46 | -.18 | -4.09 | <.001 | .03 | -8.74 | -2.98 |  |
|  | Autistic Traits | 3.42 | 1.91 | .08 | 1.79 | .073 | .01 | -0.39 | 7.40 |  |
| **Autistic Traits**  (Continuous) | **Overall Anthropomorphism** | | | | | | | | |  |
|  | Step 1 — *F* (1, 489) = 6.34, *p* = .012, *R^2^* = .013 | | | | | | | | |  |
|  | Autistic Traits | 1.30 | 0.52 | .11 | 2.52 | .012 | .01 | 0.28 | 2.39 |  |
|  | Step 2 — *F* (3, 487) = 7.90, *p* < .001, *R^2^* = .046 | | | | | | | | |  |
|  | Age | -0.12 | 0.09 | -.06 | -1.35 | .178 | .00 | -0.31 | 0.06 |  |
|  | Sex | -9.05 | 2.35 | -.18 | -3.86 | < .001 | .03 | -13.40 | -4.12 |  |
|  | Autistic Traits | 1.60 | 0.53 | .14 | 3.04 | .003 | .02 | 0.57 | 2.66 |  |
|  | **Adult Anthropomorphism** | | | | | | | | |  |
|  | Step 1 — *F* (1, 489) = 6.96, *p* = .009, *R^2^* = .014 | | | | | | | | |  |
|  | Autistic Traits | 0.72 | 0.27 | .12 | 2.64 | .009 | .01 | 0.12 | 1.31 |  |
|  | Step 2 — *F* (3, 487) = 3.96, *p* = .008, *R^2^* = .024 | | | | | | | | |  |
|  | Age | -0.02 | 0.05 | -.02 | -0.34 | .734 | .00 | -0.11 | 0.08 |  |
|  | Sex | -2.72 | 1.25 | -.10 | -2.17 | .030 | .01 | -5.00 | -0.14 |  |
|  | Autistic Traits | 0.83 | 0.28 | .14 | 2.94 | .003 | .02 | 0.20 | 1.37 |  |
|  | **Childhood Anthropomorphism** | | | | | | | | |  |
|  | Step 1 — *F* (1, 489) = 3.19, *p* = .075, *R^2^* = .006 | | | | | | | | |  |
|  | Autistic Traits | 0.58 | 0.33 | .08 | 1.79 | .075 | .01 | -0.14 | 1.25 |  |
|  | Step 2 — *F* (3, 487) = 8.66, *p* < .001, *R^2^* = .051 | | | | | | | | |  |
|  | Age | -0.11 | 0.06 | -.08 | -1.86 | .064 | .01 | -0.22 | 0.02 |  |
|  | Sex | -6.34 | 1.47 | -.19 | -4.30 | < .001 | .04 | -9.39 | -3.57 |  |
|  | Autistic Traits | 0.78 | 0.33 | .11 | 2.34 | .020 | .01 | 0.18 | 1.50 |  |

*Note*. Sex was coded as males = 1, females = 0. Autistic traits were measured using the Autism-Spectrum Quotient-10 (AQ-10), anthropomorphism was measured using the Anthropomorphism Questionnaire with scores calculated as a total score (overall) and for each subscale (adult & child). For the Autistic Traits variable, participants were categorised into the High AQ-10 = 1 or Low AQ-10 = 0 groups based on the AQ-10 cut-off. Autistic Traits as a continuous trait measure was based on AQ-10 scores between 0-10. 95% bootstrapped bias-corrected and accelerated confidence intervals (95% BCa CI) with 2000 resamples are reported.

**Supplemental Table 6**

*Multiple Regression Analyses Testing the Moderating Effect of Autistic Traits × Sex on Overall, Adult, and Childhood Anthropomorphism – Study 1*.

| Main Predictor | Model | *B* | *SE B* | *β* | *t* | *p* | *sr^2^* | 95% BCa CI | |  |
| --- | --- | --- | --- | --- | --- | --- | --- | --- | --- | --- |
|  |  |  |  |  |  |  |  | Lower | Upper |  |
| **Autistic Traits**  (Categorical) | 1. Overall Anthropomorphism — *F* (4, 486) = 4.95, *p* < .001, *R^2^* = .039 | | | | | | | | |  |
|  | Age | -0.15 | 0.09 | -.07 | -1.60 | .111 | .01 | -0.34 | 0.03 |  |
|  | Sex | -7.87 | 2.54 | -.15 | -3.10 | .002 | .02 | -12.55 | -3.09 |  |
|  | Autistic Traits | 7.85 | 3.79 | .12 | 2.07 | .039 | .01 | 0.75 | 14.76 |  |
|  | **Autistic Traits** *×* **Sex** | **-2.29** | **6.30** | **-.02** | **-0.36** | **.717** | **.00** | **-16.02** | **14.17** |  |
|  | (2) Adult Anthropomorphism — *F* (4, 486) = 2.08, *p* = .082, *R^2^* = .017 | | | | | | | | |  |
|  | Age | -0.03 | 0.05 | -.03 | -0.58 | .560 | .00 | -0.12 | 0.07 |  |
|  | Sex | -2.10 | 1.36 | -.08 | -1.55 | .122 | .01 | -4.54 | 0.34 |  |
|  | Autistic Traits | 4.05 | 2.02 | .11 | 2.00 | .046 | .01 | 0.55 | 8.08 |  |
|  | **Autistic Traits** *×* **Sex** | **-1.20** | **3.36** | **-.02** | **-0.36** | **.721** | **.00** | **-8.29** | **7.39** |  |
|  | (3) Childhood Anthropomorphism — *F* (4, 486) = 5.91, *p* < .001, *R^2^* = .046 | | | | | | | | |  |
|  | Age | -0.12 | 0.06 | -.09 | -2.05 | .041 | .01 | -0.24 | -0.00 |  |
|  | Sex | -5.77 | 1.59 | -.18 | -3.62 | <.001 | .03 | -8.77 | -2.59 |  |
|  | Autistic Traits | 3.81 | 2.38 | .09 | 1.60 | .110 | .01 | -0.78 | 8.52 |  |
|  | **Autistic Traits** *×* **Sex** | **-1.09** | **3.95** | **-.02** | **-0.28** | **.784** | **.00** | **-9.11** | **8.25** |  |
| **Autistic Traits**  (Continuous) | (1) Overall Anthropomorphism — *F* (4, 486) = 6.03, *p* < .001, *R^2^* = .047 | | | | | | | | |  |
|  | Age | -0.12 | 0.09 | -.06 | -1.35 | .176 | .00 | -0.31 | 0.07 |  |
|  | Sex | -6.12 | 4.93 | -.12 | -1.24 | .215 | .00 | -15.67 | 3.84 |  |
|  | Autistic Traits | 1.83 | 0.62 | .16 | 2.93 | .004 | .02 | 0.63 | 2.96 |  |
|  | **Autistic Traits** *×* **Sex** | **-0.78** | **1.15** | **-.07** | **-0.68** | **.498** | **.00** | **-3.14** | **1.88** |  |
|  | (2) Adult Anthropomorphism — *F* (4, 486) = 2.97, *p* = .019, *R^2^* = .024 | | | | | | | | |  |
|  | Age | -0.02 | 0.05 | -.02 | -0.34 | .733 | .00 | -0.11 | 0.08 |  |
|  | Sex | -2.33 | 2.63 | -.09 | -0.89 | .376 | .00 | -8.03 | 2.88 |  |
|  | Autistic Traits | 0.86 | 0.33 | .14 | 2.58 | .010 | .01 | 0.25 | 1.48 |  |
|  | **Autistic Traits X Sex** | **-0.10** | **0.62** | **-.02** | **-0.17** | **.869** | **.00** | **-1.37** | **1.38** |  |
|  | (3) Childhood Anthropomorphism — *F* (4, 486) = 6.71, *p* < .001, *R^2^* = .052 | | | | | | | | |  |
|  | Age | -0.11 | 0.06 | -.08 | -1.87 | .062 | .01 | -0.22 | 0.02 |  |
|  | Sex | -3.78 | 3.09 | -.12 | -1.22 | .222 | .00 | -10.10 | 2.41 |  |
|  | Autistic Traits | 0.97 | 0.39 | .13 | 2.48 | .013 | .01 | 0.20 | 1.70 |  |
|  | **Autistic Traits** *×* **Sex** | **-0.68** | **0.72** | **-.10** | **-0.94** | **.348** | **.00** | **-2.18** | **0.95** |  |

*Note*. Sex was coded as males = 1, females = 0. Autistic traits were measured using the Autism-Spectrum Quotient-10 (AQ-10), anthropomorphism was measured using the Anthropomorphism Questionnaire with scores calculated as a total score (overall) and for each subscale (adult & child). For the Autistic Traits variable, participants were categorised into the High AQ-10 = 1 or Low AQ-10 = 0 groups based on the AQ-10 cut-off. Autistic Traits as a continuous trait measure was based on AQ-10 scores between 0-10. 95% bootstrapped bias-corrected and accelerated confidence intervals (95% BCa CI) with 2000 resamples are reported. An additional power analysis indicated that we had 88% power to detect small – medium effects in this analysis (q = 0.2, α = .05). The interaction effect of each analysis is highlighted in bold font.

**Study 2**

**Supplemental Table 7**

*Factor Loadings from the EFA of the Anthropomorphism Questionnaire – Study 2.*

| Item | Factor 1 | Factor 2 |
| --- | --- | --- |
| **I sometimes wonder if my computer deliberately runs more slowly after I have shouted at it (1)** | .76 | -.14 |
| **When I was a child I always made sure my favourite toy was comfortable (e.g. sitting up or tucked into bed) when I left the room (2)** | -.01 | .77 |
| **As a child I sometimes said “hello” and “good night” to some of my favourite toys (3)** | -.11 | .81 |
| When I was a child I held birthday parties for my favourite toys (4) | -.00 | .60 |
| **On occasions I feel that my computer/printer is being deliberately awkward (5)** | .74 | -.02 |
| I sometimes wonder if my personal possessions appreciate it when I have given them a good clean (6) | .64 | .17 |
| **On occasion I feel that the weather conditions are being deliberately bad in order to ruin a social event (7)** | .65 | -.09 |
| **As a child, when I put away my toys I made sure that any odd ones lying around were placed with the others so that they wouldn’t feel lonely (8)** | -.07 | .87 |
| I do think that certain cars have a specific personality (9) | .57 | .00 |
| **If I threw out a toy when I was a child I worried that it might think I had rejected it (10)** | -.02 | .78 |
| If I accidentally break one of my favourite possessions I make sure that I apologise to it for my clumsiness (11) | .42 | .33 |
| As a child, I felt that some of my toys had become ill (12) | .28 | .47 |
| I think that some trees are friendly while others have an air of menace (13) | .54 | .11 |
| **I sometimes think that if my computer/printer is made to feel happy and/or wanted, then they will be less likely to malfunction (14)** | .82 | -.02 |
| As a child I felt at times that some of my toys were in a bad mood (15) | .37 | .42 |
| As a child, the thought of how my favourite toys would cope without me if I died was something that I worried about (16) | .27 | .44 |
| I sometimes feel that the sea can be angry (17) | .43 | .20 |
| I sometimes wonder that if toys are stored out of sight in a dark attic or room, they might feel lonely or unloved (18) | .19 | .64 |
| Part of the reason why I picked a new car/electrical item was because when I first saw it I felt that it had a friendly personality (19) | .55 | .09 |
| **When I was a child, I made sure that when I put my toys away the ones who were friends were placed side by side (20)** | .05 | .80 |

*Note. N* = 246. Anthropomorphism Questionnaire item numbers are in parentheses. 9-item Anthropomorphism Questionnaire items are in bold font. EFA = Exploratory Factor Analysis. Kaiser-Meyer-Olkin statistics were close to 1 (all values > .75), and Bartlett’s test of sphericity was significant (*p* < .001), indicating the data were suitable for EFA.

**Supplemental Table 8**

*Factor Loadings for the 9-item Anthropomorphism Questionnaire – Study 2.*

|  | EFA | | CFA (two-factor) | | CFA  (one-factor) |
| --- | --- | --- | --- | --- | --- |
| Item | Factor 1 | Factor 2 | Factor 1 | Factor 2 |  |
| I sometimes wonder if my computer deliberately runs more slowly after I have shouted at it (1) | .75 | -.04 | .74 | -- | .20 |
| On occasions I feel that my computer/printer is being deliberately awkward (5) | .80 | .03 | .81 | -- | .28 |
| On occasion I feel that the weather conditions are being deliberately bad in order to ruin a social event (7) | .60 | -.03 | .59 | -- | .16 |
| I sometimes think that if my computer/printer is made to feel happy and/or wanted, then they will be less likely to malfunction (14) | .70 | .03 | .70 | -- | .25 |
| When I was a child I always made sure my favourite toy was comfortable (e.g. sitting up or tucked into bed) when I left the room (2) | -.02 | .81 | -- | .80 | .80 |
| As a child I sometimes said “hello” and “good night” to some of my favourite toys (3) | -.03 | .81 | **--** | .79 | .79 |
| As a child, when I put away my toys I made sure that any odd ones lying around were placed with the others so that they wouldn’t feel lonely (8) | -.02 | .87 | **--** | .86 | .86 |
| If I threw out a toy when I was a child I worried that it might think I had rejected it (10) | .03 | .70 | **--** | .72 | .72 |
| When I was a child, I made sure that when I put my toys away the ones who were friends were placed side by side (20) | .06 | .78 | **--** | .80 | .80 |

*Note. N* = 492. Anthropomorphism Questionnaire item numbers are in parentheses. EFA = Exploratory Factor Analysis. CFA = Confirmatory Factor Analysis. Factor 1 = adult subscale, Factor 2 = childhood subscale. Kaiser-Meyer-Olkin statistics were close to 1 (all values > .75), and Bartlett’s test of sphericity was significant (*p* < .001), indicating the data were suitable for EFA.

**Supplemental Table 9**

*Measurement Invariance of the 9-item Anthropomorphism Questionnaire to Level of Autistic Traits using the AQ-10 Cut-off and AQ-10 Median Split, with Multi-Group Confirmatory Factor Analysis.*

| Grouping |  | χ² | df | Δχ² | CFI | ΔCFI | TLI | RMSEA | SRMR | Decision |
| --- | --- | --- | --- | --- | --- | --- | --- | --- | --- | --- |
| AQ-10  Cut-off | Configural | 169.78 | 52 | -- | .945 | -- | .923 | .096 | .038 | Accept |
|  | Metric | 176.86 | 59 | 7.08 | .945 | 0 | .932 | .090 | .040 | Accept |
|  | Scalar | 189.82 | 66 | 12.96 | .942 | -.003 | .936 | .087 | .041 | Accept |
|  | Strict | 203.22 | 75 | 13.40 | .940 | -.002 | .942 | .083 | .043 | Accept |
| AQ-10 Median Split | Configural | 162.08 | 52 | -- | .948 | -- | .928 | .093 | .036 | Accept |
|  | Metric | 174.21 | 59 | 12.13 | .946 | -.002 | .934 | .089 | .046 | Accept |
|  | Scalar | 194.85 | 66 | 20.64 | .939 | -.007 | .934 | .089 | .049 | Accept |
|  | Strict | 204.57 | 75 | 9.72 | .939 | 0 | .941 | .084 | .053 | Accept |

*Note:* χ²: chi-square goodness of fit test; df: degrees of freedom; CFI: Comparative Fit Index; TLI: Tucker-Lewis Index; RMSEA: Root Mean Squared Error of Approximation; SRMR: Standardised Root Mean Squared Residual. AQ-10 = Autism-Spectrum Quotient-10. For the AQ-10 Cut-off MCFA, participants were categorised into the High AQ-10 = 1 (*n* =62) or Low AQ-10 = 0 (*n* = 430) groups based on the AQ-10 cut-off of 6. For the Median Split MCFA, participants were split into two groups based on the AQ-10 sample median of 3: > Median = 1 (*n* = 209) or ≤ Median = 0 (*n* = 283). Invariance at the configural level is determined if fit indices are within the critical range. Invariance at the metric, scalar, and strict levels is determined if ΔCFI ≤ .01 between nested models.

**Supplemental Table 10**

*Descriptive Statistics of the 9-item Anthropomorphism Questionnaire – Study 2.*

|  | Anthropomorphism | | |
| --- | --- | --- | --- |
|  | Overall | Adult | Childhood |
| Female | 20.21(11.03) | 4.81(5.29) | 15.40(8.89) |
| Male | 15.40(11.66) | 3.92(5.00) | 11.48(8.61) |
| Low AQ-10 | 18.72(11.26) | 4.45(5.13) | 14.27(8.97) |
| High AQ-10 | 20.98(11.98) | 5.57(5.82) | 15.42(9.03) |
| Total | 19.00(11.36) | 4.59(5.23) | 14.41(8.98) |
| Score Range | 0 – 54 | 0 – 24 | 0 – 30 |

*Note.* Table reports means with standard deviations in parentheses. Autistic traits were measured using the Autism-Spectrum Quotient-10 (AQ-10). Anthropomorphism was measured using the 9-item Anthropomorphism Questionnaire and scores were calculated as an overall score, and for each subscale: adult and child. Low AQ-10 (coded as 0; 331 females, 98 males, 1 missing sex datum) = AQ-10 scores < 6. High AQ-10 (coded as 1; 38 females, 24 males) = AQ-10 ≥ 6. Sex was coded as males = 1, females = 0.

**References**

Barrett, P. (2007). Structural equation modelling: Adjudging model fit. *Personality and Individual Differences*, *42*(5), 815-824.

Ben-Shachar, M. S., Lüdecke, D., & Makowski, D. (2020). “effectsize: Estimation of Effect Size Indices and Standardized Parameters.” Journal of Open Source Software, *5*(56), 2815.

Cheung, G. W., & Rensvold, R. B. (2002). Evaluating goodness-of-fit indexes for testing measurement invariance. *Structural Equation Modeling*, *9*(2), 233-255.

Diedenhofen, B., & Musch, J. (2015). cocor: A Comprehensive Solution for the Statistical Comparison of Correlations. *PLoS ONE*, *10*(4), e0121945.

Hu, L. T., & Bentler, P. M. (1999). Cutoff criteria for fit indexes in covariance structure analysis: Conventional criteria versus new alternatives. *Structural Equation Modeling: A Multidisciplinary Journal*, *6*(1), 1-55.

Putnick, D. L., & Bornstein, M. H. (2016). Measurement invariance conventions and reporting: The state of the art and future directions for psychological research. *Developmental Review*, *41*, 71-90.

Taasoobshirazi, G., & Wang, S. (2016). The performance of the SRMR, RMSEA, CFI, and TLI: An examination of sample size, path size, and degrees of freedom. *Journal of Applied Quantitative Methods*, *11*(3), 31-39.

Waldren, L. H., Clutterbuck, R. A., & Shah, P (2021). Erroneous NICE guidance on autism screening. *The Lancet Psychiatry*, *8*(4), 276-277.
